# Structural Brain Correlations of Visuospatial and Visuoperceptual Tests in Parkinson’s Disease

**DOI:** 10.1017/S1355617717000583

**Published:** 2017-07-17

**Authors:** Anna Isabel Garcia-Diaz, Barbara Segura, Hugo Cesar Baggio, Maria Jose Marti, Francesc Valldeoriola, Yaroslau Compta, Nuria Bargallo, Carme Uribe, Anna Campabadal, Alexandra Abos, Carme Junque

**Affiliations:** 1 Department of Medicine, Faculty of Medicine and Health Science, University of Barcelona, Barcelona, Catalonia, Spain; 2 Neuroscience Institute, University of Barcelona, Barcelona, Catalonia, Spain; 3 Institut d’Investigacions Biomèdiques August Pi i Sunyer (IDIBAPS), Barcelona, Catalonia, Spain; 4 Centro de Investigación Biomédica en Red sobre Enfermedades Neurodegenerativas (CIBERNED), Hospital Clínic de Barcelona, Barcelona, Catalonia, Spain; 5 Movement Disorders Unit, Neurology Service, Hospital Clínic de Barcelona, Barcelona, Catalonia, Spain; 6 Centre de Diagnòstic per la Imatge, Hospital Clínic, Barcelona, Catalonia, Spain

**Keywords:** Degenerative disorders, Cortical thickness, Diffusion tensor imaging, Mild cognitive impairment, Neuropsychological testing

## Abstract

**Background:** Diagnosis of mild cognitive impairment in Parkinson’s disease (PD) is relevant because it is a marker for evolution to dementia. However, the selection of suitable tests to evaluate separate cognitive domains in mild cognitive impairment related to PD remains an open question. The current work aims to investigate the neuroanatomical correlates of several visuospatial/visuoperceptual tests using the same sample and a multimodal MRI approach. **Methods:** The study included 36 PD patients and 20 healthy subjects matched for age, sex, and education. The visuospatial/visuoperceptual tests selected were: Pentagon Copying Test (PCT), Judgment of Line Orientation Test (JLOT), Visual Form Discrimination Test (VFDT), Facial Recognition Test (FRT), Symbol Digit Modalities Test (SMDT), and clock copying task (CLOX2). FreeSurfer was used to assess cortical thickness, and tract-based spatial statistics was used for fractional anisotropy analysis. **Results:** Lower performance in the PCT, JLOT, and SDMT was associated with extensive cortical thickness reductions in lateral parietal and temporal regions. VFDT and CLOX2 did not show this common pattern and correlated with more limited medial occipito-temporal and occipito-parietal regions. Performance in all visuospatial/visuoperceptual tests correlated with fractional anisotropy in the corpus callosum. **Conclusions:** Our findings show that JLOT, SDMT, and PCT, in addition to differentiating patients from controls, are suitable visuospatial/visuoperceptual tests to reflect cortical thinning in lateral temporo-parietal regions in PD patients. We did not observe the dissociation between dorsal and ventral streams that was expected according to the neuropsychological classification of visuospatial and visuoperceptual tests. (*JINS*, 2018, *24*, 33–44)

## INTRODUCTION

Cognitive decline is a common non-motor manifestation of Parkinson’s disease (PD) that may start early in the course of the disease (Aarsland, Brønnick, Larsen, Tysnes, & Alves, 2009), and progresses to mild cognitive impairment (MCI) and eventually to dementia in the majority of patients (Aarsland, Andersen, Larsen, Lolk, & Kragh-Sørensen, 2003; Hely Morris, Reid, & Trafficante, 2005).

MCI in PD (PD-MCI) has been defined by the Movement Disorder Society Task Force (MDSTF) as a cognitive decline that is more severe than expected for age but with preserved functional activities (Litvan et al., 2012). According to the MDSTF guidelines, the diagnosis of PD-MCI is mainly supported by performance in five cognitive domains: attention and working memory; executive; language; memory; and visuospatial and visuoperceptual (VS/VP) functions. Among these suggested cognitive domains, visuospatial deficits have recently received particular attention because they cannot be explained by the dopaminergic imbalances seen in PD.

It is well known that several deficits in visual functions are present in different stages of PD from prodromal to dementia. In the initial stages of the degenerative process, changes in visual acuity, contrast sensitivity, and color perception have been described. An accurate assessment of these visual functions could be useful in differentiating parkinsonian symptoms (Armstrong, 2015; Weil et al., 2016). In later stages, visuospatial deficits emerge and have been associated with incident dementia in longitudinal population-based studies (Williams-Gray, Foltynie, Brayne, Robbins, & Barker, 2007; Williams-Gray et al., 2009, 2013).

Several neuropsychological tests have been suggested by the MDSTF to assess the cognitive domains stated above. This proposal assumes that the tests included in a given domain are equivalent, and, therefore, share common but not necessarily identical brain substrates.

Previous studies have addressed this issue in regards to VS/VP functions, looking for the structural correlates of several neuropsychological tests including the Facial Recognition Test (FRT), the Visual Form Discrimination Test (VFDT) (Pereira et al., 2009; Segura et al., 2014), the Judgment of Line Orientation Test (JLOT) (Filoteo, Reed, Litvan, & Harrington, 2014; Segura et al., 2014), the Pentagon Copying Test (PCT) from the Mini-Mental State Examination (MMSE) (Filoteo et al., 2014; Garcia-Diaz et al., 2014), and the clock drawing and copying tests (Pagonabarraga et al., 2013). The results obtained are discrepant probably due to the different MRI approaches, including voxel-based (Pereira et al., 2009), volumetric (Filoteo et al., 2014), and cortical thickness measures (Garcia-Diaz et al., 2014; Segura et al., 2014), as well as to heterogeneity in the PD samples used. In this context, there emerges the necessity to study the specific neuroanatomical correlates of VS/VP functions in the same sample, using common neuropsychological tests. For these purposes, we used cortical thickness as well as white matter fractional anisotropy to study five tasks classically defined as VS/VP tests (VFDT, FRT, JLOT, PCT, and the clock copying task), as well as Symbol Digit Modalities Test (SDMT), previously defined as a processing speed and attention test, but highly dependent on VS/VP processing (Lezak, Howieson, Bigler, & Tranel, 2012). A study on functional neuroimaging correlates using the oral version of the SDMT, such as that used in our study, found significant increased activation predominantly in posterior areas, specifically in the bilateral occipital cortex, cuneus, and inferior parietal regions (Forn et al., 2009).

## METHODS

### Participants

The cohort of this study was recruited from an outpatient movement disorders clinic (Parkinson’s Disease and Movement Disorders Unit, Service of Neurology, Hospital Clínic, Barcelona, Spain), and healthy subjects were recruited from the *Institut de l’Envelliment* (Barcelona, Spain). The current sample consisted of 20 healthy controls (HC) and 36 PD patients assessed between 2013 and 2015.

Inclusion criteria for patients consisted on fulfilling the diagnostic criteria for PD established by the UK PD Society Brain Bank (Daniel & Lees, 1993). Exclusion criteria for all subjects were as follows: presence of dementia according to the Movement Disorders Society criteria (Dubois et al., 2007) and clinical assessment performed by a neurologist (M.J.M., F.V., Y.C.), Hoehn and Yahr scale score >3, young-onset PD, presence of psychiatric and/or neurologic comorbidity, low global IQ score estimated by the Vocabulary subtest of the Wechsler Adult Intelligence Scale (WAIS) 3rd edition (scalar score ≤7 points), MMSE score ≤25, claustrophobia, imaging findings on MRI not compatible with PD other than mild white matter hyperintensities in the FLAIR sequence, and MRI artifacts.

Motor symptoms were assessed with the Unified Parkinson’s Disease Rating Scale, motor section (UPDRS-III) (Fahn & Elton, 1987). All PD patients were taking antiparkinsonian drugs, consisting of different combinations of L-DOPA, cathecol-O-methyltransferase inhibitors, monoamine oxidase inhibitors, dopamine agonists, and amantadine. To standardize doses, the L-DOPA equivalent daily dose (Tomlinson et al., 2010) was calculated. All assessments were done while patients were under the effect of their usual medication (*on* state).

In line with the MDSTF recommendations (Litvan et al., 2012), we assessed five cognitive domains. Attention and working memory were assessed with the Trail Making Test (TMT) (in seconds), part A (TMT A) and part B (TMT B); Digit Span Forward and Backward; the Stroop Color-Word Test and SDMT. Executive functions were evaluated with phonemic (words beginning with the letter “p” in 1 min) and semantic (animals in 1 min) fluencies. Language was assessed by the total number of correct responses in the short version of the Boston Naming Test. Memory through total learning recall (sum of correct responses from trial I to trial V), and delayed recall (total recall after 20 min) through scores on Rey’s Auditory Verbal Learning Test (RAVLT). VS/VP functions were assessed with JLOT and VFDT (see Segura et al., 2014 for the detailed protocol). This battery is recommended by the MDSTF to evaluate cognitive functions in PD and is able to detect MCI in PD (level I or level II criteria for PD-MCI, except for language, for which a single measure was used) (Litvan et al., 2012).

We divided the subjects into three groups: HC, PD patients without MCI (PD-NC), and PD patients with MCI (PD-MCI). Expected *Z* scores adjusted for age, sex, and education for each test and each subject were calculated based on a multiple regression analysis performed in the HC group (Aarsland et al., 2009). As in previous studies (Baggio et al., 2014; Segura et al., 2014), the presence of MCI was established if the *Z* score for a given test was at least 1.5 lower than the expected score in at least two tests in one domain, or in at least one test per domain in at least two domains.

Written informed consent was obtained from all study participants after full explanation of the procedures. The study was approved by the institutional Ethics Committee from the University of Barcelona (IRB00003099).

### VS/VP Assessment

The following tests were used based on their potential involvement on VS/VP functions as described in previous studies: PCT from the MMSE scored according to the Modified Mini-Mental State criteria (3MS), as described in Garcia-Diaz et al., 2014; JLOT; VFDT; FRT; the clock copying task scored according to the CLOX2 criteria (Royall, Cordes, & Polk, 1998); and SDMT (oral version) (Forn et al., 2009; Lezak et al., 2012).

### MRI Acquisition

Magnetic resonance images were acquired with a 3 Tesla scanner (MAGNETOM Trio, Siemens, Germany). The scanning protocol included high-resolution three-dimensional (3D) T1-weighted images acquired in the sagittal plane (repetition time [TR]=2300 ms, echo time [TE]=2.98 ms, inversion time [TI]=900 ms, 240 slices, field of view=256 mm; matrix size=256 × 256; 1mm isotropic voxel and an axial FLAIR sequence (TR=9000 ms, TE=96 ms). Two single-shot echo planar imaging sagittal diffusion-weighted imaging acquisitions with identical parameters (TR=7700 ms, TE=89 ms, diffusion-encoding in 30 directions at b=0 and 1000 s/mm^2^) but reversed phase-encoding direction (anterior–posterior and posterior–anterior) were obtained for each subject. These data sets were preprocessed with FSL (version 5.0.9; https://fsl.fmrib.ox.ac.uk/fsl/) tools (*topup* and *eddy*) to correct for susceptibility-related geometric distortions, eddy current distortions, and head motion. The two preprocessed images were then averaged into a single 30-direction data set, so as to increase the signal-to-noise ratio.

### Cortical Thickness Analysis

FreeSurfer software (version 5.1; available at http://surfer.nmr.harvard.edu) was used to obtain cortical thickness as previously described (Garcia-Diaz et al., 2014; Segura et al., 2014;). Comparisons between groups and regressions were assessed using a vertex-by-vertex general linear model. Different contrasts were carried out to assess differences between all study subgroups (HC *vs*. all PD patients; HC *vs*. PD-NC; HC *vs*. PD-MCI; and PD-NC *vs*. PD-MCI). Regression models included whole-brain cortical thickness as an independent factor and cognitive scores as dependent factors. To avoid clusters appearing significant purely by chance (i.e., false positives), Monte Carlo null-Z simulation with 10,000 iterations was applied to cortical thickness maps to provide clusterwise correction for multiple comparisons. Results were thresholded at a corrected *p* value of 0.05 (Hagler, Saygin, & Sereno, 2006).

### Tract-Based Spatial Statistics Analysis

Whole-brain voxelwise statistical analysis of fractional anisotropy (FA) was carried out using tract-based spatial statistics (TBSS) from FSL (Smith et al., 2006). FA images were initially created by fitting a tensor model to the averaged motion-corrected diffusion data using DTIFIT from FDT, and then brain-extracted using BET (Smith, 2002). Mean FA images were created and thinned to obtain mean FA skeletons which represent the centers of all tracts common to the group. Each subject’s aligned FA data were projected onto this skeleton and the resulting data fed into voxelwise cross-subject statistics. Voxelwise general linear model was applied using permutation-based non-parametric testing (5000 permutations) for FA analyses, correcting for multiple comparisons across space using familywise-error correction (FWE). Only clusters with FWE-corrected *p*<.05 and extension >10 voxels are reported. The JHU White-Matter Tractography Atlas was used to obtain anatomical labels of structural regions within the significant clusters.

### Statistical Analyses

Statistical analyses of demographic, neuropsychological, and structural data variables were carried out using the statistical package SPSS-20 (2011; Armonk, NY: IBM Corp.). Student’s *t* tests were used to assess group differences between PD-NC and PD-MCI. One-factor analyses of variance were used to address differences among HC, PD-NC, and PD-MCI, and Bonferroni correction was used to perform *post hoc* tests. Pearson’s chi-squared tests were applied to assess contingencies between qualitative variables. To report the effect sizes of group differences, we used Cohen’s *d* (small *d*=0.2, medium *d*=0.5 and large *d*=0.8).

## RESULTS

### Neuropsychological Performance

Demographic and clinical data of the participants are summarized in Table 1. No significant differences were found between study groups in age, sex, education, or clinical variables associated with PD.

**Table 1:** Demographic and clinical data of the participants

	HC (*n*=20)	PD (*n*=36)	*t* ^a^, χ^b^	PD-NC (*n*=26)	PD-MCI (*n*=10)	*t* ^a^, χ^b^, *F* ^c^
Age	69.15±7.94	64.37±9.97	1.855^a^	63.58±9.88	66.30±10.99	1.973^c^
Sex (male/female)	11/9	28/8	2.782^b^	20/6	8/3	3.188^b^
Education (years)	11.35±4.43	12.39±5.83	0.701^a^	13.23±5.32	11.30±6.91	0.880^c^
Age at onset		54.06±11.11		54.18±10.41	53.80±13.11	0.089^a^
Disease duration		10.342±6.01		9.42±3.99	11.90±8.66	0.870^a^
UDPRS-III		17.53±10.83		17.60±11.35	17.00±8.49	0.071^a^
H&Y		2.07±0.73		1.92±0.52	3.00±1.41	−1.072^a^
LEDD		767.63±461.53		743.15±416.84	904.70±576.21	0.935^a^

*Note.* Disease duration: duration of motor symptoms, in years; H&Y: Hoehn & Yahr; LEDD: Levodopa equivalent daily dose. Values are: mean±standard deviation.

a Student’s *t* test statistics.

b Pearson’s chi-square statistics.

c One-factor analyses of variance. No significant differences were found between study groups in age, sex, education, or clinical variables associated with PD.

Neuropsychological results are summarized in Table 2. We found significant differences in VS/VP measures between HC, PD-NC, and PD-MCI. The effect size for these differences was medium to large when the PD-MCI group was compared with their healthy peers and with PD patients without MCI (see Table 2). We did not find differences between groups in VFDT or CLOX2 performance. PCT, SDMT, and FRT showed moderate to large effect sizes when comparing PD-MCI and HC, whereas JLOT and PCT showed moderate effect sizes in the comparison between PD-MCI and PD-NC. SDMT *post hoc* testing for the comparison between PD-NC and PD-MCI did not survive Bonferroni correction (corrected *p*=.058; see Table 2).

**Table 2:** Group comparison of VS/VP performance between HC, PD-NC, and PD-MCI

	Mean±*SD*				Effect size (Cohen’s *d*)		
	HC (*n*=20)	PD-NC (*n*=26)	PD-MCI (*n*=10)	F (*p*)	HC vs PD-NC	PD-NC vs PD-MCI	HC vs PD-MCI
PCT	9.55±0.69	9.65±0.80	8.00±3.27	4.689		0.693*	0.656*
				(.013)			
JLOT	24.95±3.83	25.15±4.05	20.30±8.88	3.459		0.703*	
				(.036)			
VFDT	29.50±2.69	29.54±2.61	27.20±5.33	2.085			
				(.134)			
FRT	23.40±1.79	22.31±2.24	20.67±3.32	4.472			1.024*
				(.016)			
SDMT	46.90±10.26	45.23±12.80	32.60±22.04	3.879		0.701^**^	0.832*
				(.029)			
CLOX2	14.45±1.10	14.08±1.02	13.70±1.42	1.569			
				(.218)			

*Note.* PCT: Pentagon Copying Test scored according to the Modified Mini-Mental State criteria; CLOX2: Clock copying task scored according to CLOX2 criteria (Royall et al., 1998). F corresponds to one-factor analysis of variance. *Significant *post-hoc* analyses (*p* < .05). ^**^SDMT post-hoc analysis *p*=0.058 between PD-NC and PD-MCI.

### Cortical Thickness Comparison Between Groups

**Fig. 1:**
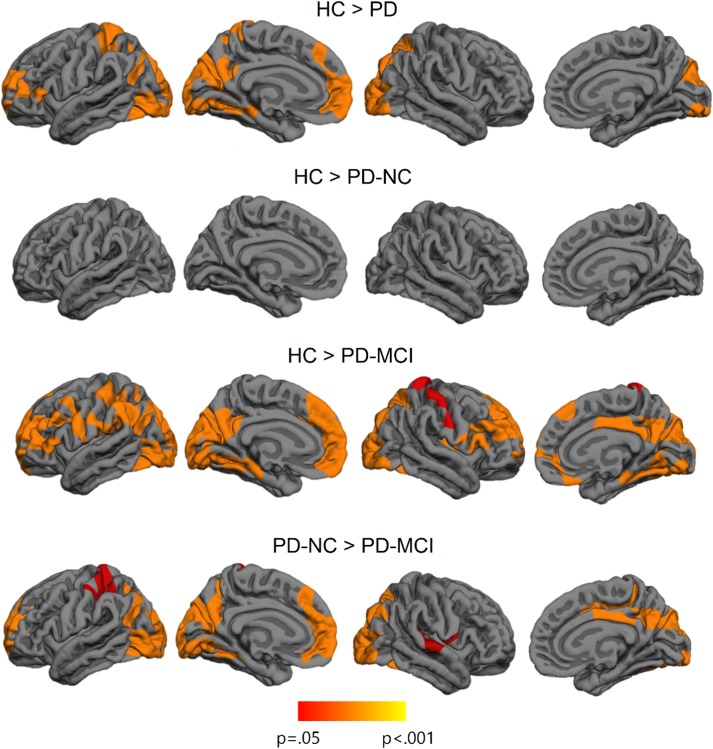
Vertex-wise cortical thickness differences between study groups. The scale bar shows *p*-values.

Imaging analyses revealed significant cortical thickness reductions in PD patients compared with healthy subjects in bilateral occipital and posterior parietal, left medial temporo-occipital, and in left frontal regions. The contrast between healthy subjects and PD-NC patients did not show significant differences. The PD-MCI group evidenced a widespread bilateral posterior–anterior pattern of cortical degeneration in comparison with the other study groups. The clusters of cortical thinning in PD-MCI were more extended when compared with healthy subjects (see Figure 1; Table 3).

**Table 3:** Significant clusters showing cortical thickness differences between HC, PD-NC, and PD-MCI.

		Talairach coordinates of the maxima				
Cluster anatomical annotation	Cluster size (mm^2^)	X	Y	Z	*Z* value	Clusterwise *p* value
HC > PD patients						
Left cuneus	14727.79	−20.1	−66.5	50.3	6.995	.0001
Left superior frontal	4682.70	−12.1	51.0	7.7	4.153	.0001
Right superior parietal	6752.04	−20.1	−55.1	50.3	3.839	.0001
HC > PD-MCI						
Left cuneus	29222.76	−13.0	−74.0	17.8	5.917	.0001
Left precuneus	1604.28	−15.1	−45.7	66.6	2.736	.0211
Right lateral occipital	12847.31	34.3	−90.5	−11.2	4.170	.0001
Right postcentral	2436.08	48.3	−16.8	49.2	3.429	.0018
Right superior frontal	7319.17	25.3	10.6	45.0	3.277	.0001
PD-NC > PD-MCI						
Left cuneus	13406.63	−20.1	−66.5	12.9	7.654	.0001
Left superior frontal	4552.57	−12.1	51.0	7.7	4.662	.0001
Left superior frontal	1475.63	−10.7	2.1	44.7	3.849	.0348
Left superior parietal	2278.55	−20.3	−40.9	60.7	3.813	.0023
Right lateral occipital	9933.19	26.8	−97.2	−10.5	4.119	.0001
Right postcentral	2221.82	53.3	−9.4	12.3	2.620	.0039

*Note.* Results were corrected using FWE correction with Monte Carlo null-Z simulation and thresholded at *p* ≤ 0.05.

### DTI Comparison Between Groups

**Fig. 2:**
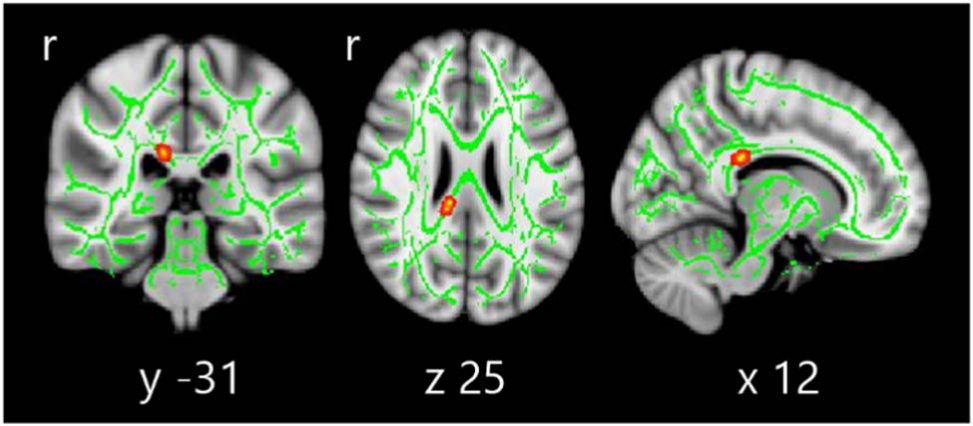
Tract-based spatial statistics differences between HC, PD-NC, and PD-MCI. Voxelwise group differences are marked in warm colors. Results are overlaid on the white matter skeleton (green) and displayed over the sagittal, coronal, and axial sections of the MNI standard brain at *p* ≤ .05 FWE-corrected. *Post hoc* analyses were significant in all group comparisons (*p* < .001).

DTI analysis revealed significant differences between study groups in FA (see Figure 2), located in the right posterior corpus callosum (coordinates of cluster maximum: X=12, Y=−31, Z=25; *p*=.017).

### Correlations Between VS/VP Tests and Cortical Thickness in PD Patients

VS/VP measures showed significant cortical thickness correlations in PD patients. The PCT correlated significantly with cortical thickness in the left lateral occipital cortex and lingual gyrus, and in bilateral temporo-parietal areas involving medial regions, such as fusiform and parahippocampal gyri, left isthmus of cingulate gyrus, and precuneus, as well as in bilateral dorsal regions, specifically superior temporal and supramarginal gyri. In addition, the PCT also correlated with thickness in anterior regions including right caudal middle frontal gyrus, bilateral precentral regions, and left anterior cingulate and superior frontal gyri.

The JLOT showed significant correlates with thickness in left lateral occipital cortex and lingual gyrus, with bilateral medial regions including left fusiform and parahippocampal gyri, bilateral precuneus, and isthmus of cingulate gyrus, as well as anterior cingulate thickness. In addition, JLOT correlated with dorsal regions including bilateral superior temporal gyrus, bilateral supramarginal gyrus, and right insula.

The SDMT correlated significantly with bilateral medial and dorsal temporo-parietal regions, including bilateral fusiform and parahippocampal gyri, bilateral precuneus, superior temporal gyri, and right supramarginal and postcentral regions. Moreover, this task correlated with an anterior medial region corresponding to the left anterior cingulate. SDMT did not correlate with cortical thickness in occipital regions except for the left lingual gyrus.

VFDT scores significantly correlated with an isolated cluster that extended to the left lingual and fusiform gyrus, whereas CLOX2 test correlated with bilateral precuneus and isthmus of cingulate gyrus.

**Fig. 3:**
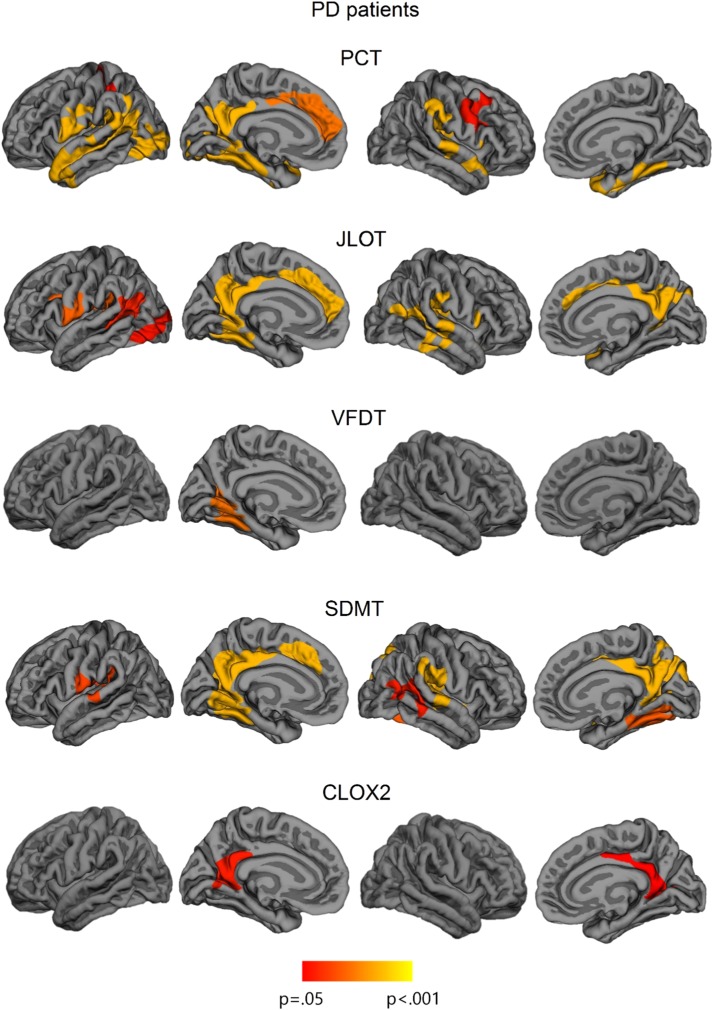
Vertex-wise cortical thickness correlations with neuropsychological measures in all sample of PD patients. The scale bar shows *p*-values. PCT: Pentagon Copying Test scored according to the Modified Mini-Mental State criteria; CLOX2: Clock copying task scored according to CLOX2 criteria (Royall et al., 1998).

Finally, there were no significant correlations between cortical thickness and FRT performance (see Figure 3; Table 4). Considering the specific role of fusiform regions in facial recognition (Haxby et al., 2001), we performed complementary intergroup and correlation analyses between FRT scores and mean fusiform gyrus cortical thickness (as defined by the Desikan-Killiany atlas) (Desikan et al., 2006). Significant intergroup effects were observed for both left fusiform (*F*=4.064; *p*=.023) and right fusiform (*F*=3.700; *p*=.031), and *post hoc* analyses showed that thickness was significantly reduced in PD-MCI compared with HC (left fusiform *p*=.023; right fusiform *p*=.043). Results of correlation analyses, however, were not statistically significant (left fusiform: *r*=0.244, *p*=.157; right fusiform: r=0.240, *p*=.164).

**Table 4:** Significant clusters showing cortical thickness correlations with VS/VP measures in the whole sample of PD patients

			Talairach coordinates of the maxima				
	Cluster anatomical annotation	Cluster size (mm^2^)	X	Y	Z	Z value	Clusterwise p value
PCT	Left precuneus	17110.87	−19.2	−56.9	17.5	4.981	0.0001
	Left superior frontal	2575.12	−12.0	44.3	5.9	3.515	0.0009
	Left superior parietal	1601.59	−33.4	−40.0	45.4	3.115	0.0308
	Right parahippocampal	6518.98	35.5	−35.3	−9.3	6.452	0.0001
	Right caudal middle frontal	1795.26	40.4	2.0	34.1	3.129	0.0239
JLOT	Left precuneus	7435.32	−18.8	−56.3	19.2	5.698	0.0001
	Left supramarginal	1977.87	−53.4	−51.4	20.8	4.182	0.0134
	Left precentral	2601.05	−47.7	4.0	13.2	3.283	0.0018
	Left lateral occipital	1770.66	−43.6	−72.4	−3.8	3.175	0.0240
	Right isthmus cingulate	3518.33	4.8	−46.8	24.4	4.262	0.0001
	Right insula	6355.79	40.1	−23.8	0.5	3.869	0.0001
VFDT	Left precuneus	2768.33	−17.7	−56.6	14.9	4.087	0.0009
SDMT	Left precuneus	7252.65	−17.1	−55.3	18.6	5.803	0.0001
	Left superior temporal	2248.37	−40.0	35.8	10.4	2.857	0.0042
	Right isthmus cingulate	4888.68	5.1	−47.2	23.8	4.828	0.0001
	Right superior temporal	4119.17	44.9	−20.9	1.2	4.223	0.0001
	Right lingual	2490.76	27.3	−44.5	−3.2	4.202	0.0022
	Right banks of superior temporal sulcus	1889.24	45.8	−39.2	12.8	2.991	0.0178
CLOX2	Left precuneus	1865.08	−26.6	−61.4	7.5	2.829	0.0176
	Right isthmus cingulate	1621.63	21.9	−50.4	7.4	3.628	0.04310

*Note.* PCT: Pentagon Copying Test scored according to the Modified Mini-Mental State criteria; CLOX2: Clock copying task scored according to the CLOX2 criteria (Royall et al., 1998). Results were corrected using FWE correction with Monte Carlo null-Z simulation and thresholded at *P*≤0.05.

In healthy subjects, SDMT and PCT showed a significant one-tailed cluster correlation with the left fusiform gyrus (*p*<.001), and PCT correlated significantly with right inferior parietal (*p*=.0001), lingual (*p*=.032), and inferior temporal regions (*p*=.033).

### Correlations Between VS/VP Tests and DTI Measures in PD Patients

**Fig. 4:**
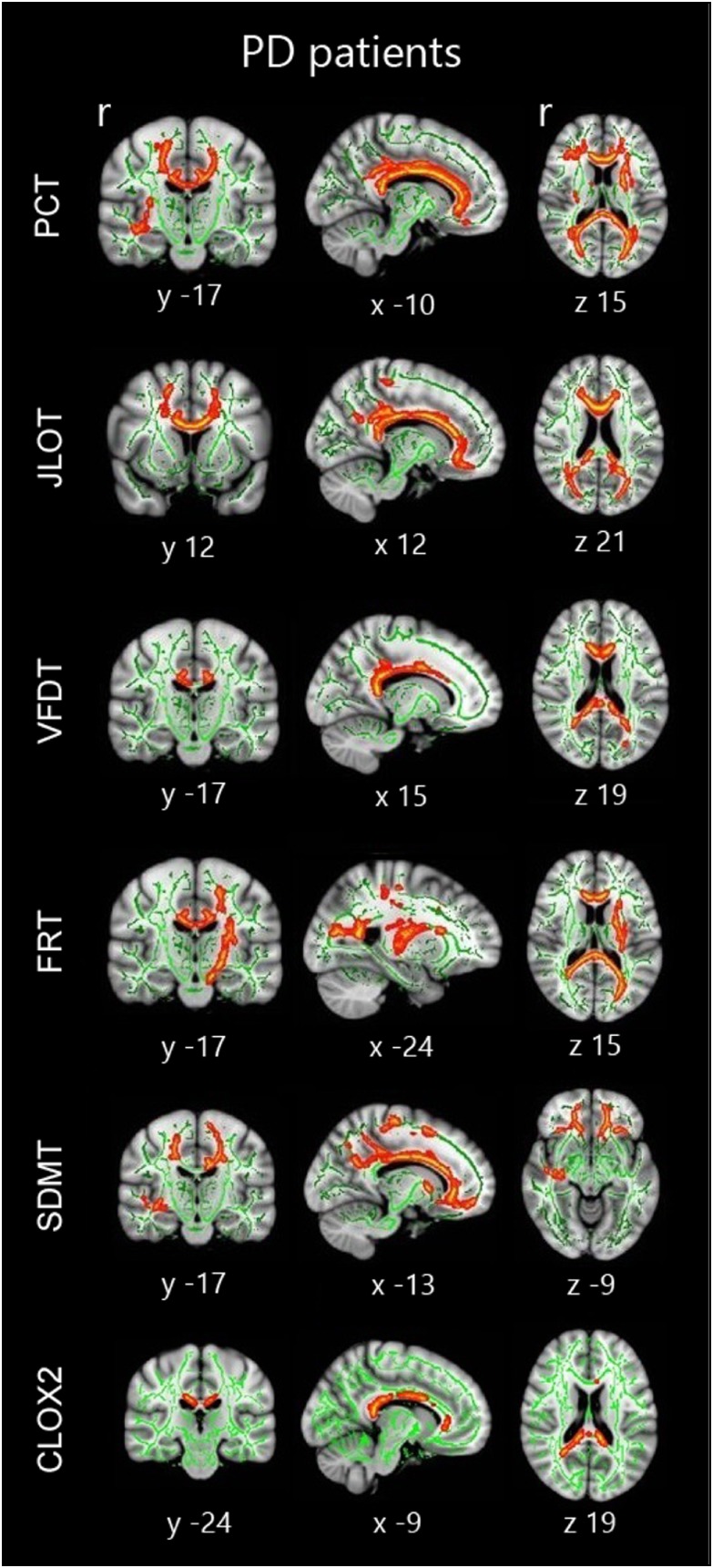
Tract-based spatial statistics correlations in the whole sample of PD patients. Significant group correlations are marked in warm colors. Results are overlaid on the white matter skeleton (green) and displayed over the sagittal, coronal and axial sections of the MNI standard brain at *p*<.05 FWE-corrected. Image shows significant clusters >10 voxels. PCT: Pentagon Copying Test scored according to the Modified Mini-Mental State criteria; CLOX2: Clock copying task scored according to the CLOX2 criteria (Royall et al., 1998).

TBSS correlation analyses revealed significant results in PD patients. All the tests studied showed significant correlations between their scores and FA values. Significant correlation in all tests included the corpus callosum, bilateral forceps minor, uncinate fasciculus, inferior fronto-occipital fasciculus, forceps major, and inferior longitudinal fasciculus. Qualitatively, among all tests, SDMT showed the most extensive correlations (see Figure 4; Table 5). In healthy subjects, there were no significant correlations between neuropsychological measures and FA values.

**Table 5:** Significant clusters showing tract-based spatial statistics correlations with neuropsychological measures in PD patients in MNI standard anatomical coordinates

	Voxels	*p v*alue at maximum	x, y, z coordinates	Hemisphere	Anatomical labels
PCT	28469	0.015	5, 24, 13	Right	Genu of corpus callosum/forceps minor
JLOT	36106	0.012	25, 34,4	Right	Inferior fronto-occipital fasciculus, uncinate fasciculus and anterior thalamic radiation
VFDT	3839	0.040	17, −41, 7	Right	Splenium of corpus callosum / forceps major
FRT	8140	0.022	12, −42, 17	Right	Splenium of corpus callosum / forceps major
	2442	0.041	−20, −4,10	Left	Posterior limb of internal capsule
SDMT	67610	0.003	9, 32, −19	Right	Forceps minor/uncinated fasciculus
CLOX2	1524	0.045	−14, −41, 8	Left	Forceps major
	93	0.049	−10, 28, −3	Left	Forceps minor

*Note.* PCT: Pentagon Copying Test scored according to the Modified Mini-Mental State criteria; CLOX2: Clock copying task scored according to the CLOX2 criteria (Royall et al., 1998). Table shows significant clusters >10 voxels at *p*<.05 FWE-corrected.

## DISCUSSION

In this study, we investigated the brain correlates of six VS/VP tests used in the diagnosis of PD-MCI in the same population, using measures from two MRI modalities: cortical thickness and FA. We evidenced specific neuroanatomical correlates of these tasks in the same sample.

Not all neuropsychological tests achieved statistical significance in the group comparisons. We found significant differences among groups for all tests except for the VFDT and CLOX2. Specifically, we observed significant differences between PD-MCI and HC in PCT, FRT and SDMT performance, whereas MCI and non-MCI patients showed differences in PCT and JLOT scores. Effect sizes were medium for all tests except for the FRT, which was large in the comparison between HC and PD-MCI.

The MDSTF guidelines on PD-MCI recommends using Benton’s JLOT, Hooper Visual Organization, and Royall’s CLOX2 to assess visuospatial functions, especially the first two tasks because of their low reliance on motor ability. We suggest that the FRT also merits consideration for the neuropsychological assessment of specific visuoperceptual functions in PD patients. Levin et al. (1991) administered six visuospatial tests to a sample of 183 patients and concluded that, in the early phases of PD, demented and non-demented patients exhibit a marked decline in FRT performance, while the impairment in JLOT performance was observed only when dementia patients were considered. Moreover, FRT has been shown to be impaired in PD with hallucinations (Ramirez-Ruiz et al., 2007).

Surprisingly, performance in the CLOX2 was similar in all groups, whereas other brief screening tests such as the PCT demonstrated significant differences between PD-MCI and the other groups, with medium effect sizes. The CLOX2 test has been reported as a useful tool in the diagnosis of dementia, but it has shown low sensitivity to detect subjects with MCI (Forti, Olivelli, Rietti, Maltoni, & Ravaglia, 2010). Regarding the VFD, a significant effect of dementia diagnosis and time of evolution has been reported (Levin et al., 1991). In our study, the exclusion of patients with dementia might, therefore, explain the lack of significant group differences in these tests.

The CLOX2 seems to be less sensitive to PD visuospatial impairment than PCT, perhaps because the former represents a more abstract and complex task. In addition, CLOX2 is influenced by semantic memory and executive functions (Cosentino, Jefferson, Chute, Kaplan, & Libon, 2004), and this association could help to compensate the VS/VP impairment in a non-demented sample. In addition, relevant longitudinal population-based studies have stated the usefulness of PCT as an incident dementia marker, probably reflecting atrophy in posterior cortical regions (Williams-Gray et al., 2007, 2009, 2013).

The patterns of cerebral correlations obtained for the VS/VP tests were also different. Lower performance in the PCT, JLOT, and SDMT were associated with cortical thickness reductions in parietal and temporal regions. The VFDT and the CLOX2, on the other hand, did not show this common pattern of correlations, and only showed spatially limited correlations with medial regions. The results obtained in the current work for the JLOT and the VFDT were similar to those obtained in our previous study assessing a larger sample (Segura et al., 2014). In that study, we studied the cortical correlates of different neuropsychological tests but we did not focus on the VS/VP domain using a neuroimaging multimodal approach. There we observed that the VFDT only correlated with cortical thickness in the right superior temporal and lingual gyri, whereas performance in the JLOT correlated with thickness in the bilateral fusiform gyri and the precuneus, as well as in the right superior temporal gyrus (Segura et al., 2014).

These results are partially in agreement with those obtained by Filoteo et al. (2014), using volumetric brain measures of specific regions of interest (ROIs), who reported significant correlations between JLOT performance and bilateral superior temporal and right lateral occipital cortices. Using voxel-based morphometry, Pereira et al. (2009) reported a positive association between VFDT performance and gray matter volumes in the fusiform gyrus, parahippocampus, middle occipital gyrus, and inferior frontal gyrus, as well as between FRT and ventral occipito-temporal cortex volume. In the present study, however, we did not find significant neuroanatomical correlates for the FRT.

Using ROI analyses, we found that PD patients and controls differed in the mean cortical thickness of the fusiform gyri and in the performance of FRT, but we did not observe significant correlations between both variables. In fact, this method is not sensitive enough to delimitate the fusiform face area (FFA) and precludes the identification of fine brain-behavior correlates. In normal subjects, the role of FFA regions in face perception was well detected by classical early fMRI studies (Haxby et al., 2001), and also by recent MRI techniques using retinotopic mapping analyses (Sangupta et al., 2016). The use of a functional MRI paradigm could have identified this specific ROI (Sengupta et al., 2016) and facilitated the correlational study between cortical atrophy and FRT performance.

The results of voxel-based morphometry and cortical thickness studies are not directly comparable because these measures are not equivalent (Pereira et al., 2012). Furthermore, the threshold for statistical significance also differed between these studies. Some previously reported results should be interpreted with caution as they were not corrected for multiple comparisons (Filoteo et al., 2014).

The PCT and the CLOX2 are both brief screening tasks that assess visuospatial functions. However, the neuroanatomical correlates of these tests are not similar. Worse performance in the PCT is related to thinning in extended medial and lateral parietal and temporal cortices, whereas performance in the CLOX2 only showed specific correlations with posterior cingulate and precuneus thickness. Pagonabarraga et al. (2013) also reported that clock-copying abilities correlated with cortical thickness in the precuneus. Different neuroanatomical correlates of these screening tools might reflect heterogeneity in the underlying deficits, or differences in sensitivity of the tests. In this sense, the PCT might more strongly reflect the impact of neurodegeneration on the visual processing system.

According to the classical model of complex visual processing (Haxby et al., 1991; Mishkin, Ungerleider, & Macko, 1983), the dorsal pathway, also called the “where” stream, is an occipito-parietal network involved in the processing of spatial information. In turn, the ventral pathway, also known as the “what” stream, is an occipito-temporal network involved in processing individual items such as faces, objects, colors, or words. According to this dissociation, we would expect to observe dorsal parietal correlates for the JLOT, CLOX2, and PCT; medial occipito-temporal correlates for the FRT; and mixed patterns for both VFDT and SDMT. However, the actual picture appears to be more complex, as there are some evidences of a trifurcation of the dorsal stream beyond the parietal cortex: the *parieto-medial temporal*, *parieto-premotor,* and the *parieto-prefrontal* pathways (Kravitz, Saleem, Baker, & Mishkin, 2011). It has been suggested that these three pathways interact with the ventral stream, and that a major point of convergence and perceptual integration is within the medial temporal lobe (see Kravitz et al., 2011 for a review).

Moreover, a recent meta-analysis on the functional MRI studies of normal subjects performing tasks specific to the “what” and “where” streams has identified specific regions according to stimulus type, and also several conjunctive regions in medial and lateral temporal cortices (Deng et al., 2016). The lack of retinotopic mapping in our sample hinders the interpretations about higher visual processing regions related to the visual tests. However, within this context, we could speculate that our results agree with the relevance of this area of interactions between pathways, given that we observed cortical thickness reductions in the medial temporal lobe related to all the studied tests, including the tasks putatively considered as visuospatial. In addition, several authors have shown reciprocal connections not only between structures such as the basal ganglia, subthalamic nucleus, and frontal cortex, but also the parietal cortex, a factor seemingly important when studying visuoperceptual functions.

White matter alterations can also play a role in cognitive impairment in PD (Hattori et al., 2012). The group comparison performed in our study showed that PD-NC and PD-MCI subjects differed in several cortical regions, but FA decreases were scarce. The modest results could be explained because DTI analyses were performed using the widely-adopted method TBSS; this method is more restrictive than whole brain voxel-wise group comparisons and projects the data onto an alignment-invariant tract representation. This approach, which initially improves the sensitivity, objectivity, and interpretability of analysis of multi-subject diffusion imaging studies, could be less sensitive to detect voxels further from tract centers or regions centered between two skeleton points (Zalesky, 2011; see Bach et al., 2014; Schwarz et al., 2014). Moreover, white matter hyperintensities and other FA-reducing abnormalities, common in elderly subjects, are also particularly problematic (Jones & Cercignani, 2010).

On the other hand, correlation analyses showed that the loss of corpus callosum integrity is related to lower cognitive performance in all VS/VP tests assessed. In addition, we also observed significant correlations in regions of the inferior fronto-occipital fasciculus. These associative fibers connect relevant regions involved in both VS/VP functions.

In this study, we focused on the neuroanatomical correlates of VS/VP functions in PD. The relatively regional degeneration of cortical structures is a factor partially explaining cognitive impairment. However, there are other functional contributors to this impairment. The dopaminergic dysfunctions affecting the basal-ganglia-thalamic-cortical dysfunctions could be in part responsible for VS/VP deficits in PD, especially considering that there are reciprocal connections not only between structures such as the basal ganglia, subthalamic nucleus and the frontal cortex, but also the parietal cortex (Seger, 2013).

On the other hand, recent evidence has revealed the contribution of functional connectivity impairment to VS/VP impairment in PD. Graph-theoretical analyses of functional networks obtained with resting-state functional MRI showed that network modularity partially explains VS/VP deficits (Baggio et al., 2014). Moreover, increased connectivity between the default mode network and both medial and lateral occipito-parietal regions in PD-MCI subjects have been shown in association with worse VS/VP performance (Baggio et al., 2015). Finally, given that our study focused on non-demented PD patients, the occurrence of a functional compensation phenomenon should be taken into consideration. This phenomenon could justify weaker structural correlates observed for several tests.

One possible limitation of our study is that, despite the inclusion of a variety of tests in the main cognitive domains defined in recent guidelines (attention and working memory, executive functions, memory, and visuospatial and visuoperceptual functions) (Litvan et al., 2012), we did not include the same number of tests in each cognitive domain, and language was assessed only with the Boston Naming Test. This may have biased the pattern of cognitive deficits present in the PD-MCI group.

Another limitation of this study was the fact that lower-level visual functions were not formally assessed. PD patients may have visual deficits that range from loss of visual acuity to complex visuoperceptual deficits. The primary visual deficits are associated with retinal dysfunctions, while the visuoperceptual deficits could be explained by the progressive degeneration of posterior brain regions (Weil et al., 2016). In our study, visual acuity was evaluated by clinical interview and review of the clinical record data of patients. The systematic assessment of visual acuity would allow us to study the contribution of this variable in the performance of complex VS/VP tests.

In summary, we found that impaired performance in the JLOT, SDMT, and PCT is related to a common pattern of cortical thinning, as well as microstructural white matter abnormalities. VFDT, CLOX2, and FRT showed less consistent and more limited results. Our findings suggest that JLOT, SDMT, and PCT are suitable tests to assess a common cognitive domain, sustained by a neuroanatomical correlate coincident with the VS/VP streams based on structural neuroimaging techniques. Further studies using longitudinal designs might be useful to shed light on the neural substrate of changes in VS/VP performance and its impact on the neurodegenerative process in PD.

The established anatomical pattern of correlation between cortical thickness measures and performance in VS/VP tests, as well as the involvement of white matter fasciculi connecting cortical regions, suggests the utility of these tests as tools to identify cognitive decline in the VS/VP domain, as well as the underlying brain network degeneration in PD.
